# Management of deep neck infection by a transnasal approach: a case report

**DOI:** 10.4076/1752-1947-3-7317

**Published:** 2009-07-31

**Authors:** Yuh Baba, Yasumasa Kato, Hideyuki Saito, Kaoru Ogawa

**Affiliations:** 1 Department of Otorhinolaryngology, Tochigi National Hospital, 1-10-37 Nakatomatsuri, Utsunomiya, Tochigi 320-8580, Japan; 2 Department of Otorhinolaryngology, Otsuka Hospital, Tokyo 152-8902, Japan; 3 Department of Otorhinolaryngology, Head and Neck Surgery, Keio University, 35 Shinanomachi Shinjuku, Tokyo 160-0082, Japan; 4 Department of Biochemistry & Molecular Biology, Kanagawa Dental College, Yokosuka 238-8580, Japan

## Abstract

**Introduction:**

Deep neck infection is a life-threatening condition, and intravenous antibiotic therapy is preferable in the early stages of the disease. However, in the advanced stages, surgical drainage should be performed. Although several surgical treatment strategies are available, it is necessary to standardize treatment according to the patient's general condition and history.

**Case presentation:**

We report the case of a 68-year-old man with a deep neck abscess and with severe diabetes mellitus and inflammation. Computed tomography identified a deep neck infection extending from the level of the epipharynx to that of the hyoid bone. We performed surgical drainage by transnasal endoscopy. The patient exhibited no evidence of either recurrent disease or post-surgical complications within 30 months of follow-up.

**Conclusions:**

This case report provides evidence that transnasal endoscopic drainage should be recommended as a standard approach in patients with a deep neck abscess and with a severe general condition, diabetes mellitus, and inflammation.

## Introduction

Deep neck infection is a life-threatening condition with various serious complications, such as, airway obstruction, cranial nerve palsy, descending necrotizing mediastinitis, internal carotid compression, and rupture [1]. Its localization on the floor of the mouth can be a particularly serious threat. The etiology of a deep neck infection can be varied. Parhiscar and Har-El determined the etiology, location, and bacteriology in 210 cases. The most common causes of a deep neck abscess were dental infection (43%) and intravenous drug abuse (12%). About 70% of the abscesses were in two locations, the submandibular space and the lateral pharyngeal space; and the most frequent bacteria responsible for abscess formation were *Streptococcus viridans, Staphylococcus epidermidis, Staphylococcus aureus*, and β-hemolytic streptococci [2]. Diabetes mellitus (DM) was a common associated systemic disease occurring in 34 of the 210 cases (16%) [2]. They also reported that the incidence of abscesses in the retropharyngeal space was 12% (25/210 cases), including eight cases (32%) treated with tracheotomy [2].

In selected cases where the extent of the infection is limited, conservative treatment with intravenous antibiotic therapy can be successful. In advanced cases, however, surgical exploration with drainage of the abscess is generally required. Different traditional surgical approaches have been described in relation to the site of infection and the involvement of adjacent structures [3].

Endoscopic approaches have a number of important advantages in comparison to external approaches, including minimal complication, the absence of cervical scarring, and a short operation time. Nagy *et al.* reported successful treatment of 22/23 pediatric patients by transoral drainage of deep neck infections, including three cases of parapharyngeal abscess [4]. Transnasal endoscopic drainage for retroparapharyngeal abscess was reported in two cases [5].

We report that transnasal endoscopy can be effective for the drainage of deep neck abscesses in patients in poor general condition, such as those with severe DM and inflammation.

## Case presentation

**Figure 1:**
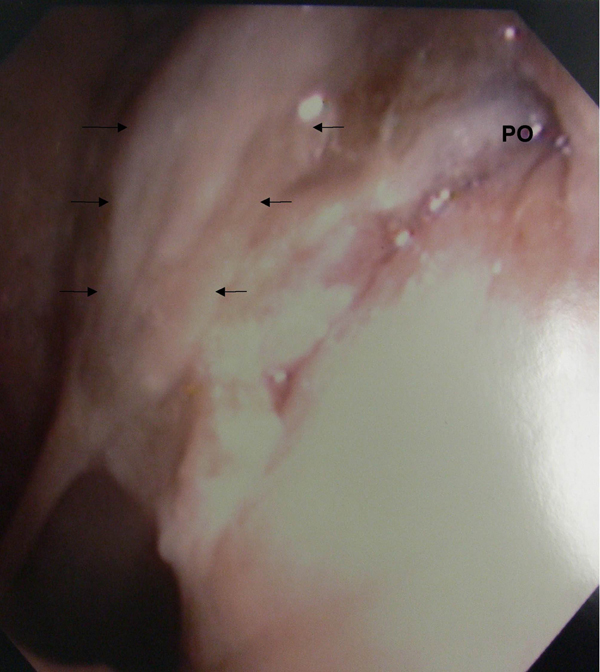
**Transnasal endoscopic view of the left nasal cavity showing left lateral nasopharyngeal wall edema (black arrow) and the pharyngeal opening of the auditory tube (PO)**.

**Figure 2:**
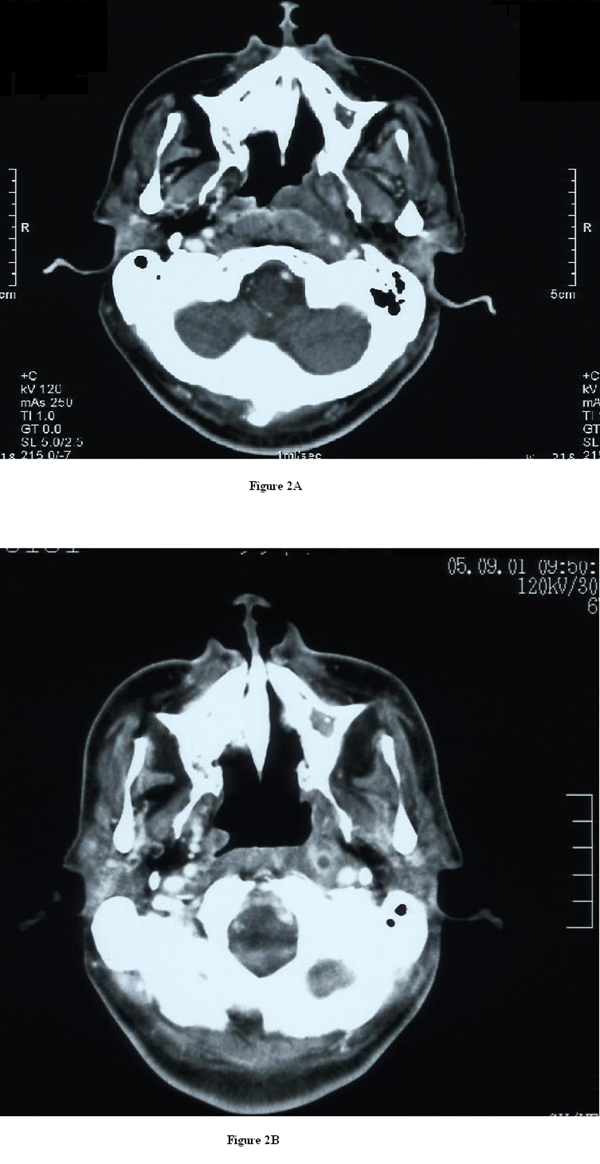
****(A)** Computed tomography of the neck with intravenous contrast showing the left cellulitis at the level of the nasopharynx (white arrow)**. **(B)** Computed tomography examination (6 weeks after surgery) indicated the presence of a necrotic retropharyngeal lymph node at the level of the nasopharynx (white arrow).

In July 2005, a 68-year-old man was admitted to our institution with a 3-week history of low-grade fever and headache on the left side. The patient had a history of chronic sinusitis, and also had DM. Transnasal fiberscopy revealed left lateral pharyngeal wall edema, or asymmetrical bulging from the level of the Rosenmüller fossa to that of the uvula, and no inflammatory signs in the pharyngeal tonsil (Figure 1). Laboratory evaluation revealed severe inflammatory conditions in our patient and suggested the requirement for surgical treatment: white blood cells (WBC), 19,000 cells/mm^3^; C-reactive protein (CRP), 42.68 mg/dL; HbA1c, 14.5%; and glucose, 565 mg/dL. Computed tomography (CT) of the neck showed marked thickening of the epipharynx on the left side (Figure 2A). The lesion extended from the level of the epipharynx to that of the hyoid bone. Signs of left hypoglossal palsy and left Horner's syndrome were also evident. Thus, the lesion was due to deep neck infection extending from the epipharynx to the surrounding poststyloid space, although CT did not show any typical features of abscess at this time (1 week before the operation) (Figure 2A). Micro-otoscopy showed left purulent otorrhea through a central perforation of the tympanic membrane.

As symptoms persisted despite antibiotic therapy (meropenem trihydrate 1 g twice/day and clindamycin 600 mg twice/day), we suspected a very acute stage of abscess formation after diagnosis by CT. Unfortunately, we could not repeat the CT scan to confirm abscess formation, because repeated CT scans to check progress within 30 days are not covered by either Employees' Insurance or National Health Insurance in the Japanese Social Insurance System. We elected not to perform a transcervical procedure under general anesthesia due to severe DM, severe inflammation, and trismus. In addition, an external approach under local anesthesia was considered difficult because the region of suspected abscess formation was located mainly between the levels of the base of the skull and soft palate medial to the great vessels, and did not involve multiple spaces, and the patient did not have any airway obstruction. Therefore, due to the patient's severe general condition and the anatomical location of the lesion, we instead chose to perform surgical drainage using a transnasal endoscopic approach under local anesthesia 1 week after CT scanning.

A vertical incision, 1 cm in length, was made in the lateral epipharyngeal wall. The opening was enlarged by removing some inflamed mucosa with forceps. We observed no apparent pharyngeal constrictor muscle on transnasal endoscopic surgery, which may have been due to severe tissue encroachment by inflammation, and we could reach into the poststyloid space using only forceps without a microdebrider. Dissection into the poststyloid space produced an enormous amount of purulent material. Upon inspection with a 30° angled fiberscope, pulsation of the left internal carotid artery was clearly visible. The operation time was 20 minutes. Cultures of the purulent material yielded *S. aureus*. Histological examination revealed only nonspecific inflammatory cells and fibrous cells. These clinical and laboratory observations supported our diagnosis of deep neck abscess.

**Figure 3:**
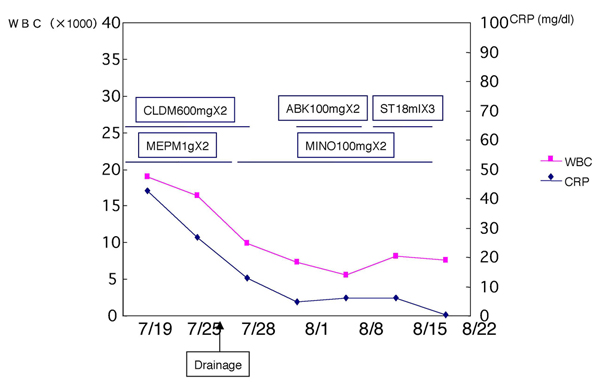
**Treatment process**. CLDM, clindamycin; MEPM, meropenem trihydrate; ABK, arbekacin sulfate; ST, sulfamethoxazole trimethoprim; MINO, minocycline hydrochloride.

The postoperative course was uneventful. Transnasal endoscopic aspiration was started on the first postoperative day and continued once a day for 30 days. The patient reported a rapid improvement in symptoms, except those related to cranial nerve palsy. Six weeks later, endoscopy showed normalization of the epipharynx. CT examination demonstrated the presence of a small necrotic retropharyngeal lymph node at the level of the epipharynx (Figure 2B). Laboratory analyses showed improvement in inflammation: WBC, 7600 cells/mm^3^; CRP, 0.47 mg/dL (Figure 3). Thirty months after surgery, the patient had no symptoms other than those related to cranial nerve palsy.

## Discussion

Although the advancement of antibiotics has markedly reduced the incidence and mortality rates, deep neck infection remains a challenging problem due to the complex anatomy and potentially lethal complications that may arise [1]. Deep neck infection is usually due to odontogenic, pharyngeal, tonsillar, salivary gland, middle ear, or mastoid infections [2,6]. In this patient, we observed otitis media from the middle ear but no signs of odontogenic, pharyngeal, tonsillar, or salivary gland inflammation. We could not determine the exact sequence of the events leading to the onset of deep neck infection in this patient, whether the acute otitis media was secondary to compression of the eustachian tube or vice versa, or whether the deep neck infection was a complication of a middle ear infection. As the patient suffered repeatedly from acute sinusitis 3 months after the operation, we postulated that the deep neck infection occurred following acute sinusitis.

Appropriate treatment planning for patients with a deep neck infection requires clear differentiation between cellulitis and abscess. Imaging of the soft tissue of the neck has developed significantly using CT scanning technology, which plays a fundamental role in the diagnosis of deep neck infection. In this patient, CT showed cellulitis, but no apparent abscess one week before the operation. However, we observed an enormous amount of purulent material on dissection into the poststyloid space. This discrepancy may have been because the patient was in a very acute stage of abscess development from cellulitis seven days after diagnosis based on the results of the CT scan.

In addition to external incision for drainage, percutaneous ultrasound- or CT-guided aspiration of deep neck abscesses using a spinal needle has been reported [7,8]. However, we could not use these approaches in this patient due to a lack of typical imaging features of the abscess on the CT scan. In addition, the patient's general condition was poor due to severe DM and inflammation, and therefore a minimally invasive treatment was required. Thus, a transnasal endoscopic approach, which could reveal the lesion by visual inspection, was advantageous in this patient.

Several surgical approaches are available in relation to the site and extent of the infection [3]. Deep neck infections are usually drained through an external approach. As recommended by Sethi and Stanley [9], the entire cavity is then explored by blunt finger dissection to avoid any residual purulent material, particularly in the case of multilocular abscesses. Moreover, tracheotomy can be performed to avoid respiratory distress in patients with compromised upper airway patency.

In this patient, the abscess was located mainly in the upper region from the epipharynx to the surrounding poststyloid space near the great vessels, and the possibility of draining the collection by an endoscopic transnasal route, without resorting to an external approach, was offered to this patient.

To our knowledge, there have only been two previous reports of a transnasal endoscopic approach for drainage of a deep neck abscess [5,10]. Sethi and Stanley briefly mentioned the use of a transnasal endoscopic approach in eight patients with deep neck infections, without providing information about the indications or surgical technique used [10]. Nicolai *et al.* reported that the main surgical steps were incision of the pharyngeal mucosal wall with a diode laser, widening of the incision by eliminating some inflamed mucosa with a microdebrider, drainage of purulent collection, and careful dissection and removal of the necrotic tissue [5].

Generally, external drainage requires about 2 to 3 hours. The transnasal endoscopic approach involves a shorter operating time than the external approach with minimal complications. However, transnasal endoscopic drainage for a deep neck abscess is also accompanied by a risk of a relatively long hospitalization period depending on the requirement for repetition of drainage. With regard to both key issues, the transnasal endoscopic approach was considered suitable especially in elderly patients with severe concomitant diseases. Here, we present a patient with a deep neck abscess treated successfully by transnasal endoscopy as the patient's general condition was considered unsuitable for general anesthesia (because of factors including age, DM, severe inflammation and trismus). The lesion was located mainly from the level of the base of the skull to that of the soft palate, located medial to the great vessels, and did not involve multiple spaces. The patient did not have airway obstruction. To date, only a small number of patients have been treated by the transnasal endoscopic approach. Endoscopic approaches have a number of important advantages in comparison to external approaches, including minimal complications, the absence of cervical scarring, availability of repeated drainage, and short operation time. Therefore, transnasal endoscopic drainage of a deep neck abscess is an effective alternative to external approaches in patients with a severe general condition, DM, and inflammation. Despite these advantages, however, it is still controversial as to whether the transnasal endoscopic approach for treatment of deep neck abscesses is suitable for patients with a better general condition as this approach is associated with a risk of vascular injury, dyspnea, and incomplete drainage.

## Conclusions

The observations in our patient indicated that transnasal endoscopic drainage has advantages for treatment of deep neck abscesses in patients with a severe general condition, diabetes mellitus, and inflammation. Thus, this method could be recommended as a standard approach in such cases.

## Abbreviations

CRP: C-reactive protein; CT: computed tomography; DM: diabetes mellitus; WBC: white blood cells.

## Consent

Written informed consent was obtained from the patient for publication of this case report and any accompanying images. A copy of the written consent is available for review by the Editor-in-Chief of this journal.

## Competing interests

The authors declare that they have no competing interests.

## Authors' contributions

YB assisted in the conception and design of the paper, and also helped in the acquisition, review and interpretation of the data. YK and HS contributed towards data collection and drafting of the manuscript. KO was involved in conception, reviewing and finally approving the version to be published. All authors read and approved the final manuscript.

## References

[B1] Sichel JY Dano I Hocwald E Biron A Eliashar R Nonsurgical management of parapharyngeal space infections: a prospective study Laryngoscope 2002 112 906 910 10.1097/00005537-200205000-00023 12150626

[B2] Parhiscar A Har-El G Deep neck abscess: a retrospective review of 210 cases Ann Otol Rhinol Laryngol 2001 110 1051 1054 11713917 10.1177/000348940111001111

[B3] Toshima M Deep neck infection Antibiot Chemother (Japanese) 2000 16 1715 1720

[B4] Nagy M Pizzuto M Backstrom J Brodsky L Deep neck infections in children: a new approach to diagnosis and treatment Laryngoscope 1997 107 1627 1634 10.1097/00005537-199712000-00010 9396677

[B5] Nicolai P Lombardi D Berlucchi M Farina D Zanetti D Drainage of retro-parapharyngeal abscess: an additional indication for endoscopic sinus surgery Eur Arch Otorhinolaryngol 2005 262 722 730 10.1007/s00405-004-0890-1 15668811

[B6] Lazor JB Cunningham MJ Eavey RD Weber AL Comparison of computed tomography and surgical findings in deep neck infections Otolaryngol Head Neck Surg 1994 111 746 750 10.1016/S0194-5998(94)70562-3 7991254

[B7] Baatenburg de Jong RJ Rongen RJ Lameris JS Knegt P Verwoerd CD Ultrasound-guided percutaneous drainage of deep neck abscesses Clin Otolaryngol Allied Sci 1990 15 159 166 10.1111/j.1365-2273.1990.tb00450.x 2190716

[B8] Poe LB Petro GR Matta I Percutaneous CT-guided aspiration of deep neck abscesses Am J Neuroradiol 1996 17 1359 1363 8871725 PMC8338523

[B9] Sethi DS Stanley RE Parapharyngeal abscesses J Laryngol Otol 1991 105 1025 1030 10.1017/S0022215100118122 1787355

[B10] Sethi DS Stanley RE Deep neck abscesses-changing trends J Laryngol Otol 1994 108 138 143 10.1017/S0022215100126106 8163915

